# 6-Amino-3-methyl-4-(3,4,5-tri­meth­oxy­phen­yl)-2,4-di­hydro­pyrano[2,3-*c*]pyrazole-5-carbo­nitrile

**DOI:** 10.1107/S1600536814015670

**Published:** 2014-07-23

**Authors:** Naresh Sharma, Goutam Brahmachari, Bubun Banerjee, Rajni Kant, Vivek K. Gupta

**Affiliations:** a Post-Graduate Department of Physics & Electronics, University of Jammu, Jammu Tawi 180 006, India; b Laboratory of Natural Products & Organic Synthesis, Department of Chemistry, Visva-Bharati University, Santiniketan 731 235, West Bengal, India

**Keywords:** crystal structure

## Abstract

In the title compound, C_17_H_18_N_4_O_4_, the dihedral angle between the benzene ring and 2,4-di­hydro­pyrano[2,3-*c*]pyrazole ring system is 89.41 (7)°. The pyran moiety adopts a strongly flattened boat conformation. In the crystal, mol­ecules are linked by N—H⋯N, N—H⋯O, C—H⋯N and C—H⋯O hydrogen bonds into an infinite two-dimensional network parallel to (110). There are π–π inter­actions between the pyrazole rings in neighbouring layers [centroid–centroid distance = 3.621 (1) Å].

## Related literature   

For background to the biological activity of synthetic pyrano[2,3-*c*] pyrazole compounds, see: Zaki *et al.* (2006 ▶); Abdelrazek *et al.* (2007 ▶); Mohamed *et al.* (2010 ▶); Bhavanarushi *et al.* (2013 ▶). For the synthesis of the title compound, see: Brahmachari & Banerjee (2014 ▶). For a related structure, see: Low *et al.* (2004 ▶).
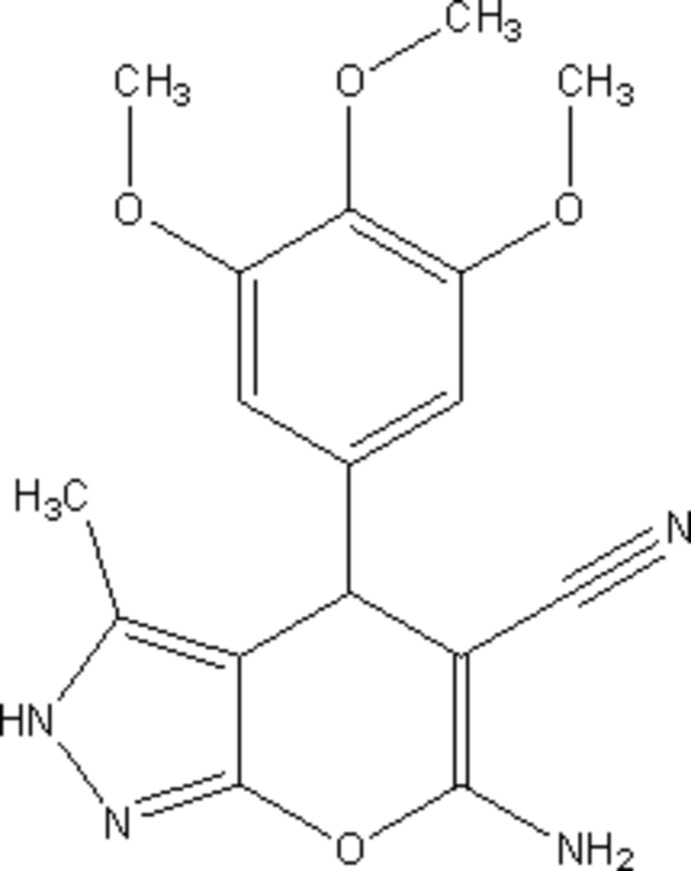


## Experimental   

### 

#### Crystal data  

C_17_H_18_N_4_O_4_
*M* *_r_* = 342.35
Triclinic, 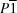

*a* = 7.6168 (6) Å
*b* = 9.9967 (5) Å
*c* = 11.7888 (6) Å
α = 105.283 (5)°
β = 99.416 (5)°
γ = 92.221 (5)°
*V* = 851.05 (9) Å^3^
*Z* = 2
Mo *K*α radiation
μ = 0.10 mm^−1^
*T* = 293 K
0.30 × 0.20 × 0.20 mm

#### Data collection  

Oxford Diffraction Xcalibur Sapphire3 diffractometer
Absorption correction: multi-scan (*CrysAlis PRO*; Oxford Diffraction, 2010 ▶) *T*_min_ = 0.805, *T*_max_ = 1.000
6228 measured reflections
3344 independent reflections
2201 reflections with *I* > 2σ(*I*)
*R*_int_ = 0.030

#### Refinement  

*R*[*F* ^2^ > 2σ(*F* ^2^)] = 0.047
*wR*(*F* ^2^) = 0.125
*S* = 1.00
3344 reflections
242 parameters
H atoms treated by a mixture of independent and constrained refinement
Δρ_max_ = 0.22 e Å^−3^
Δρ_min_ = −0.22 e Å^−3^

### 

Data collection: *CrysAlis PRO* (Oxford Diffraction, 2010 ▶); cell refinement: *CrysAlis PRO*; data reduction: *CrysAlis PRO*; program(s) used to solve structure: *SHELXS97* (Sheldrick, 2008 ▶); program(s) used to refine structure: *SHELXL97* (Sheldrick, 2008 ▶); molecular graphics: *ORTEP-3 for Windows* (Farrugia, 2012 ▶); software used to prepare material for publication: *PLATON* (Spek, 2009 ▶).

## Supplementary Material

Crystal structure: contains datablock(s) I, New_Global_Publ_Block. DOI: 10.1107/S1600536814015670/gk2612sup1.cif

Structure factors: contains datablock(s) I. DOI: 10.1107/S1600536814015670/gk2612Isup2.hkl

Supporting information file. DOI: 10.1107/S1600536814015670/gk2612Isup3.cml

CCDC reference: 973484

Additional supporting information:  crystallographic information; 3D view; checkCIF report

## Figures and Tables

**Table 1:** Hydrogen-bond geometry (Å, °) *Cg*3 is the centroid of the phenyl ring.

*D*—H⋯*A*	*D*—H	H⋯*A*	*D*⋯*A*	*D*—H⋯*A*
N2—H30⋯O20^i^	0.94 (2)	1.96 (2)	2.882 (2)	165 (2)
N11—H40⋯N1^ii^	0.95 (2)	2.11 (2)	3.030 (3)	163 (2)
N11—H50⋯N10^iii^	0.91 (2)	2.25 (2)	3.156 (3)	172 (2)
C8—H8*C*⋯O18^i^	0.96	2.52	3.305 (3)	139
C19—H19*B*⋯N1^iv^	0.96	2.52	3.455 (4)	165
C21—H21*A*⋯*Cg*3^v^	0.96	2.85	3.55	130

Symmetry codes: (i) 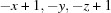; (ii) 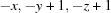; (iii) 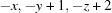; (iv) 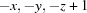; (v) 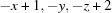.

**Fig. 1.**
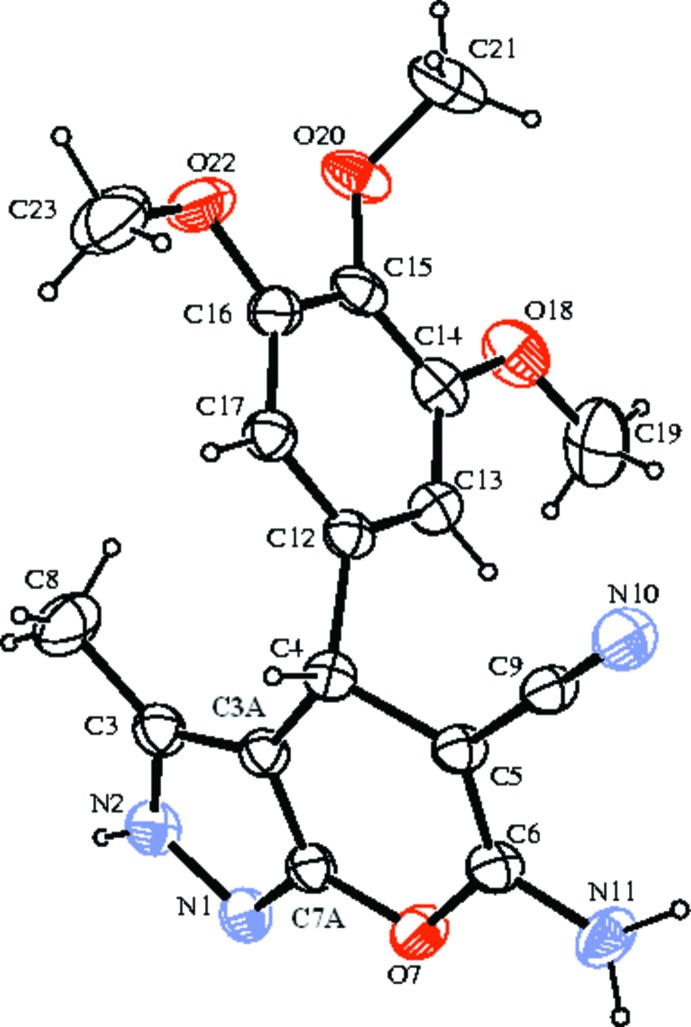
ORTEP view of the molecule with the atom-labeling scheme. The displacement ellipsoids are drawn at the 40% probability level. H atoms are shown as small spheres of arbitrary radii.

**Fig. 2.**
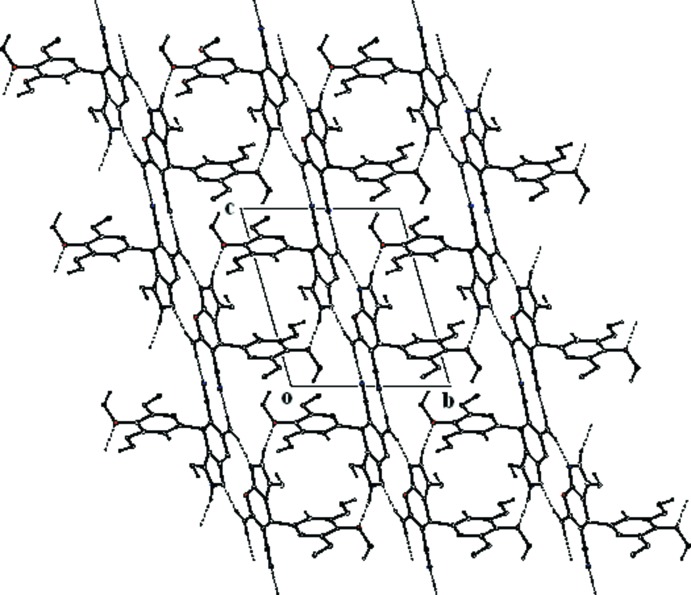
The packing arrangement of molecules viewed down the a axis.

## supplementary crystallographic information

### S1. Comment

Pyrano[2,3-*c*]pyrazole scaffolds represent a "privileged" structural motif well distributed in bioactive natural products and pharmaceutically potent synthetic heterocycles possessing a wide range of activities (Abdelrazek *et al.*, 2007; Zaki *et al.*, 2006; Mohamed *et al.*, 2010; Bhavanarushi *et al.*, 2013). Hence, investigation of the structural features of biologically relevant pyrano[2,3-*c*]pyrazole derivatives is of both scientific and practical interest. In continuation of our efforts to develop useful synthetic protocols for biologically significant molecules, we herein report an efficient and environmentally benign synthesis and the crystal structure of the title compound. In this communication we wish to report the crystal structure of 6-amino-3-methyl-4-(3,4,5-trimethoxyphenyl)-2, 4-dihydropyrano[2,3-*c*]pyrazole-5-carbonitrile (1) synthesized *via* one-pot multicomponent reaction (MCR) at room temperature using commercially available urea as inexpensive and environmentally benign organo-catalyst. The structure of the title compound 1 was elucidated by spectral methods and X-ray diffraction studies. The bond distances in the title compound are comparable to the closely related structure (Low *et al.*, 2004). In the title compound, C_17_H_18_N_4_O_4_, the dihedral angle between the benzene ring (C12/C13/C14/C15/C16/C17) and the pyrazole ring is 87.05 (7)° and between pyrano and pyrazole rings is 4.69 (8)°. The benzene ring and the pyrazole ring are nearly planar with a maximum deviation of 0.0018 Å for the phenyl C3 atom and 0.0227 Å for the pyrazole C15 atom. The pyran moiety adopts a strongly flattened boat conformation with one mirror plane passing through the atoms C4 and O7 and the other bisecting the bonds C3A—C7A and C5—C6. In the crystal, molecules are linked by N—H···N hydrogen bonds into infinite two-dimensional network parallel to (110) . There are π - π interactions between the pyrazole rings in neighbouring networks [centroid–centroid seperation = 3.621 (1) Å, interplanar spacing = 3.333 Å, centriod shift = 1.242 Å, symmetry code: 1 - *x*,1 - *y*,1 - *z*].

### S2. Experimental

The synthesis of the title compound was carried out *via* one-pot multi-component reaction in aqueous ethanol using low-cost and environmentally benign urea as catalyst at room temperature. An oven-dried screw cap test tube was charged with a magnetic stir bar, ethyl acetoacetate (0.130 g, 1.0 mmol) and hydrazine hydrate (0.050 g, 1 mmol). The reaction mixture was then stirred at room temperature for about 10 min and 3,4,5-trimethoxybenzaldehyde (0.196 g, 1 mmol), malononitrile (0.066 g, 1.1 mmol), urea (0.007 g, 10 mol % as organo-catalyst) and EtOH:H2O (1:1 *v*/*v*; 4 ml) were added in a sequential manner (Brahmachari & Banerjee, 2014). The reaction mixture was then stirred vigorously at room temperature and the stirring was continued for 16 h. The progress of the reaction was monitored by TLC. On completion of the reaction, a solid was precipitated out, filtered off and repeatedly washed with aqueous ethanol to obtain a crude product which was purified by recrystallization from ethanol without carrying out column chromatography. The structure of the title compound was confirmed by analytical as well as spectral studies, including ^1^H NMR, ^13^C NMR, and TOF-MS. Single crystal was obtained from DMSO. For crystallization 50 mg of compound was dissolved in 5 ml DMSO and left for several days at ambient temperature.

6-Amino-3-methyl-4-(3,4,5-trimethoxyphenyl)-2,4-dihydropyrano[2,3-*c*] pyrazole-5-carbonitrile (1). White solid. Yield 89%. Mp: 491–493 K. ^1^H NMR (400 MHz, DMSO-d_6_) δ /p.p.m.: 12.11(1*H*, s, NH), 6.87 (2*H*, s, NH_2_), 6.47 (2*H*, s, aromatic H), 4.59 (1*H*, s, CH), 3.72 (6*H*, s, 2 \ OCH_3_), 3.64 (3*H*, s, OCH_3_), 1.87 (3*H*, s, CH_3_). ^13^C NMR (100 MHz, DMSO-d_6_) δ /p.p.m.: 161.39, 155.11, 153.20 (2 C), 140.49, 136.55, 136.20, 121.29, 104.98 (2 C), 97.74, 60.38, 57.33, 56.22 (2 C), 36.87, 10.34. TOF-MS: 365.1230 [*M*+Na]+. Elemental analysis: Calcd. (%) for C_17_H_18_N_4_O_4_: C, 59.64; H, 5.30; N, 16.37; found: C, 59.62; H, 5.28; N, 16.39.

### S3. Refinement

The positions of the N-H group H atoms were determined from a difference Fourier map and freely refined. All the remaining H atoms were placed geometrically and allowed to ride on their parent C atoms, with C—H distances of 0.93–0.98 Å; and with *U*_iso_(H) = 1.2*U*_eq_(C), except for the methyl group where *U*_iso_(H) = 1.5*U*_eq_(C).

### Crystal data

**Table:**

C_17_H_18_N_4_O_4_	*Z* = 2
*M**_r_* = 342.35	*F*(000) = 360
Triclinic, *P*1	*D*_x_ = 1.336 Mg m^−^^3^
Hall symbol: -P 1	Mo *K*α radiation, λ = 0.71073 Å
*a* = 7.6168 (6) Å	Cell parameters from 1789 reflections
*b* = 9.9967 (5) Å	θ = 4.1–26.7°
*c* = 11.7888 (6) Å	µ = 0.10 mm^−^^1^
α = 105.283 (5)°	*T* = 293 K
β = 99.416 (5)°	Block, colourless
γ = 92.221 (5)°	0.30 × 0.20 × 0.20 mm
*V* = 851.05 (9) Å^3^	

### Data collection

**Table:**

Oxford Diffraction Xcalibur Sapphire3 diffractometer	3344 independent reflections
Radiation source: fine-focus sealed tube	2201 reflections with *I* > 2σ(*I*)
Graphite monochromator	*R*_int_ = 0.030
Detector resolution: 16.1049 pixels mm^-1^	θ_max_ = 26.0°, θ_min_ = 3.5°
ω scans	*h* = −9→4
Absorption correction: multi-scan (*CrysAlis PRO*; Oxford Diffraction, 2010)	*k* = −12→12
*T*_min_ = 0.805, *T*_max_ = 1.000	*l* = −14→14
6228 measured reflections	

### Refinement

**Table:**

Refinement on *F*^2^	Primary atom site location: structure-invariant direct methods
Least-squares matrix: full	Secondary atom site location: difference Fourier map
*R*[*F*^2^ > 2σ(*F*^2^)] = 0.047	Hydrogen site location: inferred from neighbouring sites
*wR*(*F*^2^) = 0.125	H atoms treated by a mixture of independent and constrained refinement
*S* = 1.00	*w* = 1/[σ^2^(*F*_o_^2^) + (0.0518*P*)^2^] where *P* = (*F*_o_^2^ + 2*F*_c_^2^)/3
3344 reflections	(Δ/σ)_max_ < 0.001
242 parameters	Δρ_max_ = 0.22 e Å^−^^3^
0 restraints	Δρ_min_ = −0.22 e Å^−^^3^

### Special details

**Table:**

Experimental. *CrysAlis PRO*, Agilent Technologies, Version 1.171.36.28 (release 01–02-2013 CrysAlis171. NET) (compiled Feb 1 2013,16:14:44) Empirical absorption correction using spherical harmonics, implemented in SCALE3 ABSPACK scaling algorithm.
Geometry. All e.s.d.'s (except the e.s.d. in the dihedral angle between two l.s. planes) are estimated using the full covariance matrix. The cell e.s.d.'s are taken into account individually in the estimation of e.s.d.'s in distances, angles and torsion angles; correlations between e.s.d.'s in cell parameters are only used when they are defined by crystal symmetry. An approximate (isotropic) treatment of cell e.s.d.'s is used for estimating e.s.d.'s involving l.s. planes.
Refinement. Refinement of *F*^2^ against ALL reflections. The weighted *R*-factor *wR* and goodness of fit *S* are based on *F*^2^, conventional *R*-factors *R* are based on *F*, with *F* set to zero for negative *F*^2^. The threshold expression of *F*^2^ > σ(*F*^2^) is used only for calculating *R*-factors(gt) *etc*. and is not relevant to the choice of reflections for refinement. *R*-factors based on *F*^2^ are statistically about twice as large as those based on *F*, and *R*- factors based on ALL data will be even larger.

### Fractional atomic coordinates and isotropic or equivalent isotropic displacement parameters (Å^2^)

**Table:**

	*x*	*y*	*z*	*U*_iso_*/*U*_eq_	
N1	0.2660 (2)	0.39361 (17)	0.45176 (13)	0.0399 (4)	
N2	0.4190 (2)	0.32739 (18)	0.44265 (15)	0.0420 (4)	
C3	0.5002 (3)	0.3075 (2)	0.54703 (17)	0.0403 (5)	
C3A	0.3973 (3)	0.36369 (18)	0.63060 (16)	0.0332 (5)	
C4	0.4095 (3)	0.37099 (18)	0.76047 (15)	0.0329 (4)	
H4	0.5116	0.4362	0.8064	0.039*	
C5	0.2393 (3)	0.43016 (19)	0.79658 (15)	0.0340 (5)	
C6	0.1121 (3)	0.4790 (2)	0.72636 (16)	0.0377 (5)	
O7	0.11958 (19)	0.47720 (15)	0.61077 (11)	0.0469 (4)	
C7A	0.2595 (3)	0.4131 (2)	0.56623 (16)	0.0353 (5)	
C8	0.6670 (3)	0.2363 (3)	0.5545 (2)	0.0644 (7)	
H8A	0.6381	0.1384	0.5413	0.097*	
H8B	0.7392	0.2738	0.6322	0.097*	
H8C	0.7317	0.2505	0.4947	0.097*	
C9	0.2164 (3)	0.44234 (19)	0.91550 (16)	0.0354 (5)	
N10	0.1998 (2)	0.45179 (18)	1.01250 (14)	0.0487 (5)	
N11	−0.0360 (3)	0.5354 (2)	0.75602 (17)	0.0532 (5)	
C12	0.4390 (3)	0.22862 (19)	0.78092 (15)	0.0329 (5)	
C13	0.3050 (3)	0.1214 (2)	0.73981 (16)	0.0393 (5)	
H13	0.1912	0.1378	0.7066	0.047*	
C14	0.3410 (3)	−0.0109 (2)	0.74832 (16)	0.0413 (5)	
C15	0.5115 (3)	−0.03574 (19)	0.79687 (16)	0.0394 (5)	
C16	0.6430 (3)	0.0735 (2)	0.84271 (17)	0.0387 (5)	
C17	0.6066 (3)	0.20604 (19)	0.83509 (16)	0.0363 (5)	
H17	0.6945	0.2797	0.8663	0.044*	
O18	0.2197 (2)	−0.12489 (15)	0.71128 (13)	0.0599 (5)	
C19	0.0428 (4)	−0.1075 (3)	0.6622 (3)	0.0823 (9)	
H19A	−0.0082	−0.0431	0.7213	0.123*	
H19B	−0.0270	−0.1956	0.6379	0.123*	
H19C	0.0435	−0.0718	0.5942	0.123*	
O20	0.5514 (2)	−0.17034 (13)	0.79545 (11)	0.0522 (4)	
C21	0.5144 (4)	−0.2128 (2)	0.89635 (19)	0.0656 (8)	
H21A	0.5801	−0.1499	0.9684	0.098*	
H21B	0.5494	−0.3052	0.8905	0.098*	
H21C	0.3889	−0.2117	0.8979	0.098*	
O22	0.8041 (2)	0.04130 (15)	0.89463 (14)	0.0568 (4)	
C23	0.9389 (3)	0.1526 (3)	0.9471 (3)	0.0761 (8)	
H23A	0.9602	0.1989	0.8882	0.114*	
H23B	1.0471	0.1167	0.9763	0.114*	
H23C	0.9006	0.2176	1.0122	0.114*	
H40	−0.119 (3)	0.569 (2)	0.703 (2)	0.072 (8)*	
H30	0.442 (3)	0.290 (2)	0.365 (2)	0.063 (7)*	
H50	−0.072 (3)	0.543 (2)	0.8274 (19)	0.055 (6)*	

### Atomic displacement parameters (Å^2^)

**Table:**

	*U*^11^	*U*^22^	*U*^33^	*U*^12^	*U*^13^	*U*^23^
N1	0.0455 (11)	0.0444 (11)	0.0312 (9)	0.0059 (8)	0.0110 (8)	0.0099 (7)
N2	0.0508 (12)	0.0445 (11)	0.0330 (9)	0.0095 (8)	0.0158 (9)	0.0091 (8)
C3	0.0465 (13)	0.0395 (12)	0.0386 (11)	0.0069 (9)	0.0128 (10)	0.0132 (9)
C3A	0.0377 (12)	0.0316 (11)	0.0321 (10)	0.0048 (8)	0.0098 (9)	0.0091 (8)
C4	0.0360 (11)	0.0307 (11)	0.0313 (10)	0.0023 (8)	0.0046 (8)	0.0082 (8)
C5	0.0399 (12)	0.0357 (11)	0.0270 (9)	0.0085 (9)	0.0056 (9)	0.0090 (8)
C6	0.0469 (13)	0.0412 (12)	0.0275 (10)	0.0104 (9)	0.0088 (9)	0.0114 (9)
O7	0.0494 (9)	0.0688 (11)	0.0315 (7)	0.0247 (8)	0.0135 (7)	0.0226 (7)
C7A	0.0393 (12)	0.0383 (12)	0.0321 (10)	0.0065 (9)	0.0127 (9)	0.0120 (9)
C8	0.0692 (18)	0.0812 (19)	0.0544 (14)	0.0381 (15)	0.0274 (13)	0.0243 (13)
C9	0.0389 (12)	0.0350 (11)	0.0328 (11)	0.0116 (8)	0.0059 (9)	0.0094 (9)
N10	0.0560 (13)	0.0601 (13)	0.0333 (10)	0.0164 (9)	0.0119 (9)	0.0144 (9)
N11	0.0534 (13)	0.0800 (15)	0.0365 (10)	0.0348 (11)	0.0180 (10)	0.0236 (10)
C12	0.0405 (12)	0.0333 (11)	0.0262 (9)	0.0066 (9)	0.0092 (9)	0.0078 (8)
C13	0.0405 (12)	0.0394 (12)	0.0380 (11)	0.0050 (9)	0.0081 (9)	0.0097 (9)
C14	0.0549 (14)	0.0334 (12)	0.0341 (11)	−0.0029 (10)	0.0125 (10)	0.0049 (9)
C15	0.0609 (15)	0.0302 (11)	0.0328 (10)	0.0104 (10)	0.0199 (10)	0.0106 (9)
C16	0.0448 (13)	0.0408 (12)	0.0357 (10)	0.0137 (10)	0.0132 (9)	0.0143 (9)
C17	0.0402 (12)	0.0342 (11)	0.0352 (10)	0.0036 (9)	0.0084 (9)	0.0101 (9)
O18	0.0702 (12)	0.0415 (10)	0.0623 (10)	−0.0144 (8)	0.0072 (9)	0.0098 (8)
C19	0.0618 (19)	0.0663 (19)	0.103 (2)	−0.0226 (14)	0.0191 (17)	−0.0026 (16)
O20	0.0901 (13)	0.0325 (8)	0.0418 (8)	0.0183 (8)	0.0266 (8)	0.0130 (7)
C21	0.111 (2)	0.0492 (15)	0.0487 (13)	0.0143 (14)	0.0246 (14)	0.0269 (12)
O22	0.0504 (10)	0.0523 (10)	0.0726 (11)	0.0182 (8)	0.0060 (8)	0.0266 (9)
C23	0.0474 (16)	0.0711 (19)	0.106 (2)	0.0087 (13)	−0.0095 (15)	0.0304 (16)

### Geometric parameters (Å, º)

**Table:**

N1—C7A	1.321 (2)	C12—C17	1.386 (2)
N1—N2	1.368 (2)	C13—C14	1.388 (3)
N2—C3	1.351 (2)	C13—H13	0.9300
N2—H30	0.94 (2)	C14—O18	1.366 (2)
C3—C3A	1.377 (3)	C14—C15	1.390 (3)
C3—C8	1.481 (3)	C15—C16	1.384 (3)
C3A—C7A	1.378 (3)	C15—O20	1.387 (2)
C3A—C4	1.501 (2)	C16—O22	1.369 (2)
C4—C5	1.523 (3)	C16—C17	1.387 (3)
C4—C12	1.524 (2)	C17—H17	0.9300
C4—H4	0.9800	O18—C19	1.413 (3)
C5—C6	1.360 (2)	C19—H19A	0.9600
C5—C9	1.415 (2)	C19—H19B	0.9600
C6—N11	1.336 (3)	C19—H19C	0.9600
C6—O7	1.369 (2)	O20—C21	1.429 (2)
O7—C7A	1.371 (2)	C21—H21A	0.9600
C8—H8A	0.9600	C21—H21B	0.9600
C8—H8B	0.9600	C21—H21C	0.9600
C8—H8C	0.9600	O22—C23	1.420 (3)
C9—N10	1.151 (2)	C23—H23A	0.9600
N11—H40	0.95 (2)	C23—H23B	0.9600
N11—H50	0.91 (2)	C23—H23C	0.9600
C12—C13	1.380 (3)		
			
C7A—N1—N2	101.46 (15)	C17—C12—C4	118.71 (16)
C3—N2—N1	113.28 (17)	C12—C13—C14	119.61 (19)
C3—N2—H30	129.5 (14)	C12—C13—H13	120.2
N1—N2—H30	116.4 (14)	C14—C13—H13	120.2
N2—C3—C3A	106.36 (18)	O18—C14—C13	125.0 (2)
N2—C3—C8	121.06 (19)	O18—C14—C15	114.81 (18)
C3A—C3—C8	132.59 (18)	C13—C14—C15	120.19 (18)
C3—C3A—C7A	103.61 (16)	C16—C15—O20	120.11 (19)
C3—C3A—C4	133.13 (18)	C16—C15—C14	119.79 (17)
C7A—C3A—C4	123.20 (18)	O20—C15—C14	120.06 (17)
C3A—C4—C5	106.47 (15)	O22—C16—C15	115.96 (17)
C3A—C4—C12	110.49 (15)	O22—C16—C17	124.12 (18)
C5—C4—C12	114.14 (14)	C15—C16—C17	119.92 (19)
C3A—C4—H4	108.5	C12—C17—C16	119.95 (18)
C5—C4—H4	108.5	C12—C17—H17	120.0
C12—C4—H4	108.5	C16—C17—H17	120.0
C6—C5—C9	117.19 (18)	C14—O18—C19	118.08 (18)
C6—C5—C4	125.60 (16)	O18—C19—H19A	109.5
C9—C5—C4	117.11 (15)	O18—C19—H19B	109.5
N11—C6—C5	127.32 (18)	H19A—C19—H19B	109.5
N11—C6—O7	109.26 (16)	O18—C19—H19C	109.5
C5—C6—O7	123.42 (19)	H19A—C19—H19C	109.5
C6—O7—C7A	115.23 (15)	H19B—C19—H19C	109.5
N1—C7A—O7	119.07 (17)	C15—O20—C21	114.32 (16)
N1—C7A—C3A	115.30 (18)	O20—C21—H21A	109.5
O7—C7A—C3A	125.62 (16)	O20—C21—H21B	109.5
C3—C8—H8A	109.5	H21A—C21—H21B	109.5
C3—C8—H8B	109.5	O20—C21—H21C	109.5
H8A—C8—H8B	109.5	H21A—C21—H21C	109.5
C3—C8—H8C	109.5	H21B—C21—H21C	109.5
H8A—C8—H8C	109.5	C16—O22—C23	117.11 (17)
H8B—C8—H8C	109.5	O22—C23—H23A	109.5
N10—C9—C5	179.2 (2)	O22—C23—H23B	109.5
C6—N11—H40	122.9 (15)	H23A—C23—H23B	109.5
C6—N11—H50	124.7 (13)	O22—C23—H23C	109.5
H40—N11—H50	112.3 (19)	H23A—C23—H23C	109.5
C13—C12—C17	120.38 (17)	H23B—C23—H23C	109.5
C13—C12—C4	120.79 (17)		
			
C7A—N1—N2—C3	0.3 (2)	C4—C3A—C7A—O7	1.5 (3)
N1—N2—C3—C3A	−0.3 (2)	C3A—C4—C12—C13	−69.8 (2)
N1—N2—C3—C8	179.5 (2)	C5—C4—C12—C13	50.1 (2)
N2—C3—C3A—C7A	0.2 (2)	C3A—C4—C12—C17	106.20 (19)
C8—C3—C3A—C7A	−179.6 (2)	C5—C4—C12—C17	−133.86 (17)
N2—C3—C3A—C4	177.34 (19)	C17—C12—C13—C14	−2.8 (3)
C8—C3—C3A—C4	−2.4 (4)	C4—C12—C13—C14	173.21 (16)
C3—C3A—C4—C5	−172.4 (2)	C12—C13—C14—O18	179.75 (17)
C7A—C3A—C4—C5	4.2 (2)	C12—C13—C14—C15	−0.6 (3)
C3—C3A—C4—C12	−48.0 (3)	O18—C14—C15—C16	−176.85 (16)
C7A—C3A—C4—C12	128.68 (19)	C13—C14—C15—C16	3.5 (3)
C3A—C4—C5—C6	−5.9 (2)	O18—C14—C15—O20	5.5 (3)
C12—C4—C5—C6	−128.09 (19)	C13—C14—C15—O20	−174.18 (15)
C3A—C4—C5—C9	177.73 (15)	O20—C15—C16—O22	−5.8 (3)
C12—C4—C5—C9	55.6 (2)	C14—C15—C16—O22	176.56 (17)
C9—C5—C6—N11	−1.5 (3)	O20—C15—C16—C17	174.73 (16)
C4—C5—C6—N11	−177.8 (2)	C14—C15—C16—C17	−3.0 (3)
C9—C5—C6—O7	178.23 (17)	C13—C12—C17—C16	3.3 (3)
C4—C5—C6—O7	1.9 (3)	C4—C12—C17—C16	−172.74 (16)
N11—C6—O7—C7A	−175.81 (17)	O22—C16—C17—C12	−179.90 (17)
C5—C6—O7—C7A	4.4 (3)	C15—C16—C17—C12	−0.4 (3)
N2—N1—C7A—O7	−179.27 (16)	C13—C14—O18—C19	−1.1 (3)
N2—N1—C7A—C3A	−0.2 (2)	C15—C14—O18—C19	179.25 (19)
C6—O7—C7A—N1	172.79 (16)	C16—C15—O20—C21	94.3 (2)
C6—O7—C7A—C3A	−6.2 (3)	C14—C15—O20—C21	−88.0 (2)
C3—C3A—C7A—N1	0.0 (2)	C15—C16—O22—C23	−177.29 (19)
C4—C3A—C7A—N1	−177.51 (16)	C17—C16—O22—C23	2.2 (3)
C3—C3A—C7A—O7	179.01 (18)		

### Hydrogen-bond geometry (Å, º)

Cg3 is the centroid of the phenyl ring.

**Table:**

*D*—H···*A*	*D*—H	H···*A*	*D*···*A*	*D*—H···*A*
N2—H30···O20^i^	0.94 (2)	1.96 (2)	2.882 (2)	165 (2)
N11—H40···N1^ii^	0.95 (2)	2.11 (2)	3.030 (3)	163 (2)
N11—H50···N10^iii^	0.91 (2)	2.25 (2)	3.156 (3)	172 (2)
C8—H8*C*···O18^i^	0.96	2.52	3.305 (3)	139
C19—H19*B*···N1^iv^	0.96	2.52	3.455 (4)	165
C21—H21*A*···*Cg*3^v^	0.96	2.85	3.55	130

Symmetry codes: (i) −*x*+1, −*y*, −*z*+1; (ii) −*x*, −*y*+1, −*z*+1; (iii) −*x*, −*y*+1, −*z*+2; (iv) −*x*, −*y*, −*z*+1; (v) −*x*+1, −*y*, −*z*+2.

## References

[bb1] Abdelrazek, F. M., Metz, P., Kataeva, O., Jager, A. & El-Mahrouky, S. F. (2007). *Arch. Pharm.* **340**, 543–548. 10.1002/ardp.200700157 17912679

[bb2] Bhavanarushi, S., Kanakaiah, V., Yakaiah, E., Saddanapu, V., Addlagatta, A. & Rani, V. J. (2013). *Med. Chem. Res.* **22**, 2446–2454.

[bb3] Brahmachari, G. & Banerjee, B. (2014). *ACS Sustainable Chem. Eng.* **2**, 411–422.

[bb4] Farrugia, L. J. (2012). *J. Appl. Cryst.* **45**, 849–854.

[bb5] Low, J. N., Cobo, J., Portilla, J., Quiroga, J. & Glidewell, C. (2004). *Acta Cryst.* E**60**, o1034–o1037. 10.1107/S0108270104015616 15295199

[bb6] Mohamed, N. R., Khaireldin, N. Y., Fahmy, A. F. & El-Sayed, A. A. (2010). *Der Pharma Chem* **2**, 400–417.

[bb7] Oxford Diffraction (2010). *CrysAlis PRO* Oxford Diffraction Ltd, Yarnton, England.

[bb8] Sheldrick, G. M. (2008). *Acta Cryst.* A**64**, 112–122. 10.1107/S0108767307043930 18156677

[bb9] Spek, A. L. (2009). *Acta Cryst.* D**65**, 148–155. 10.1107/S090744490804362X PMC2631630 19171970

[bb10] Zaki, M. E. A., Soliman, H. A., Hiekal, O. A. & Rashad, A. E. Z. (2006). Z. *Naturforsch. Teil C*, **61**, 1–5. 10.1515/znc-2006-1-201 16610208

